# Translation, cultural adaptation, and psychometric validation of the Female Sexual Function Index to Lebanese Arabic (FSFI-LB)

**DOI:** 10.1093/sexmed/qfaf055

**Published:** 2025-08-05

**Authors:** Stephanie Zakhour, Walter Gonçalves, Hugo Santos, Douglas Rodrigues, Nadine El Kassis, David Atallah, Antonio Egídio Nardi, Aline Sardinha

**Affiliations:** Sexuality and its Disorders Unit, Institute of Psychiatry, Federal University of Rio de Janeiro (UFRJ), Rio de Janeiro, RJ 22290-140, Brazil; Sexuality and its Disorders Unit, Institute of Psychiatry, Federal University of Rio de Janeiro (UFRJ), Rio de Janeiro, RJ 22290-140, Brazil; Department of Statistics, Federal University of Rio Grande do Sul (UFRGS), Porto Alegre, Rio Grande do Sul, RS 90010-150, Brazil; Department of Statistics, Fluminense Federal University (UFF), São Domingos, Niterói, RJ 24210200 , Brazil; Department of Statistics, Fluminense Federal University (UFF), São Domingos, Niterói, RJ 24210200 , Brazil; Department of Gynecology and Abnormal Placentation, Hotel Dieu de France, Université Saint Joseph Beyrouth, Alfred Naccache Boulevard, Beirut, Lebanon; Department of Gynecology and Abnormal Placentation, Hotel Dieu de France, Université Saint Joseph Beyrouth, Alfred Naccache Boulevard, Beirut, Lebanon; Sexuality and its Disorders Unit, Institute of Psychiatry, Federal University of Rio de Janeiro (UFRJ), Rio de Janeiro, RJ 22290-140, Brazil; Sexuality and its Disorders Unit, Institute of Psychiatry, Federal University of Rio de Janeiro (UFRJ), Rio de Janeiro, RJ 22290-140, Brazil

**Keywords:** sexual health, culturally competent care, surveys and questionnaires, Arab world, Lebanon

## Abstract

**Background:**

The Female Sexual Function Index (FSFI) is widely used to assess female sexual function, but its applicability in Lebanon is limited due to linguistic and cultural barriers.

**Aim:**

This study aimed to develop and validate a Lebanese Arabic version of the FSFI (FSFI-LB) to improve accessibility for clinical and research use.

**Methods:**

A study was conducted with 119 Lebanese women between April 2023 and June 2024 at Hôtel-Dieu de France Hospital, Beirut. The FSFI was translated, back-translated, and psychometrically validated using exploratory factor analysis, Cronbach’s alpha, test–retest reliability, and factor correlations.

**Outcomes:**

The FSFI-LB showed excellent internal consistency (Cronbach’s alpha = 0.973) and test–retest reliability (*r* = 0.9997), with a Kaiser–Meyer–Olkin value of 0.925 and significant Bartlett’s test of sphericity (*P* < .001). Test–retest reliability was assessed in a subsample of 42 participants after 4 weeks and demonstrated high consistency across all domains (*r* > 0.99) and total scale reliability (*r* = 0.9997).

**Results:**

A six-factor model was retained for the FSFI-LB, aligning with the original FSFI, with the pain domain showing the weakest correlations with other domains. The questionnaire included English translations alongside Arabic terms for improved comprehension.

**Clinical Translation:**

The FSFI-LB is a reliable tool for assessing female sexual function in Lebanon, ensuring cultural and linguistic sensitivity for diverse populations.

**Strengths and Limitations:**

The study’s strengths include robust psychometric validation and the adaptation of a widely used instrument. The limitations include the focus on a specific Lebanese population, which may limit generalizability to other Arabic-speaking communities.

**Conclusion:**

The FSFI-LB is a valid and reliable tool for assessing female sexual function, contributing to the development of culturally appropriate measures in sexual health research and clinical practice.

## Introduction

Female sexual health is a multidimensional aspect of well-being shaped by complex interactions between physiological, psychological, and clinical factors, as well as sociocultural influences.^1^ However, research on the clinical assessment and treatment of female sexual dysfunction (FSD) in Lebanon remains scarce,^2^ with limited diagnostic tools and therapeutic strategies to address sexual health concerns in diverse cultural contexts. Structural barriers—including cultural stigma, social norms, and the absence of standardized, culturally sensitive screening tools—hinder Lebanese women’s ability to seek and receive adequate clinical support.^3^ In addition, gender and sexuality-related taboos compromise professional training among healthcare and mental health practitioners,^4^ restricting their ability to effectively assess and treat sexual health issues. One study found that only 31% of Lebanese obstetricians/gynecologists (Ob/Gyns) proactively discuss sexual health with their patients.^5^ These challenges highlight the urgent need for clinically validated and culturally relevant tools to enhance sexual health assessment, therapy, and intervention strategies.

The Female Sexual Function Index (FSFI) is a widely used self-report instrument designed to assess female sexual function across six domains: desire, arousal, lubrication, orgasm, sexual satisfaction, and pain.^6^ Despite its broad use, its applicability in clinical and research settings remains limited in Lebanon due to the lack of a validated, linguistically accessible version. While an FSFI adaptation exists for Arabic conducted in Egypt,^7^ it is based on a distinct dialect and terminology that may not be fully comprehensible to Lebanese patients, potentially affecting diagnostic accuracy and self-reported outcomes.^3^^,^^8^ Lebanon’s linguistic diversity, influenced by French, English, and Levantine Arabic, further complicates the standardization of medical terminology in sexual health assessments. Many Lebanese women have limited fluency in Classical Arabic medical terminology, which may impact their ability to accurately report symptoms and engage in clinical discussions.^9^ Moreover, the Egyptian version includes a cultural and demographic focus that may not accurately reflect the diversity of experiences in Lebanon, such as the inclusion of only Muslim women, married women, and women who had female genital mutilation (FGM), which differs from the Lebanese context. Given the high prevalence of FSD in Arabic-speaking populations, an FSFI adaptation that maintains strong psychometric properties and clinical utility is essential to support accurate assessments, effective interventions, and improved patient-reported outcomes. This study addresses this gap by developing and validating the FSFI in Lebanese Arabic (FSFI-LB), ensuring its reliability, diagnostic precision, and applicability for both clinical and research use.

In response to the lack of validated instruments for assessing female sexual function in Lebanon, and in line with previous clinically adapted research,^10^^,^^11^ this study aims to provide a clinically applicable and psychometrically robust translation and validation of the FSFI in Lebanese Arabic (FSFI-LB). By ensuring high reliability, diagnostic accuracy, and clinical utility, the FSFI-LB offers healthcare professionals, sex therapists, and researchers a standardized tool to assess FSD in both clinical and research settings. This validation supports evidence-based sexual health assessments, enhances diagnostic precision, and facilitates the development of targeted interventions for sexual dysfunction. Given the journal’s emphasis on multidisciplinary research in sexual medicine, this study contributes to the advancement of standardized outcome measures, ultimately improving patient care and sexual health research in diverse populations.

## Methods

### Study design and setting

This cross-sectional study conducted between April 2023 and June 2024 followed established guidelines for the translation, cultural adaptation, and psychometric validation of the scale.^12^ It was conducted at the gynecology department clinic of Hôtel-Dieu de France Hospital (HDF) in Beirut, Lebanon. Ethical approval was obtained from the hospital’s ethics committee (#CEHDF1997) before initiating the study. Data collection included both clinic-based and online participation to broaden the sample reach, especially given the sensitivity of the topic.

### Recruitment of participants

Participants were recruited using convenience sampling at the clinic and the snowball technique, where initial participants referred others, to increase reach and participation.^13^ Given the cultural sensitivity of the topic and the stigma surrounding discussions of sexual health in Lebanon, this method allowed us to access a broader pool of participants beyond those attending the clinic. Women were encouraged to refer friends or relatives who might be interested in participating. This approach was particularly helpful as the clinic is in Beirut, where cultural taboos are often heightened, especially among the predominantly Muslim population. Interested participants were contacted by phone to explain the study design, objectives, and procedures clearly. It was emphasized that participation was voluntary and that they could withdraw at any time from the study. Those who agreed to participate signed informed consent forms prior to their inclusion in the study. Surveys were administered online via SurveyMonkey (www.surveymonkey.com), enabling women outside the clinic to join the study while ensuring privacy and ease of participation.

### Inclusion and exclusion criteria

Women eligible to participate in the study were aged between 18 and 55 years to minimize the inclusion of postmenopausal individuals, as hormonal changes can influence sexual function. Participants were required to have engaged in sexual activity within the last 4 weeks, as the FSFI evaluates sexual function during this period. They also needed to be nonpregnant, as pregnancy can affect sexual health, and have Lebanese nationality to ensure cultural and linguistic relevance. Additionally, participants had to be able to read Arabic and provide informed consent, and they should not have chronic or serious mental illnesses, which could affect sexual function.

### Data collection

The sociodemographic questionnaire gathered detailed participant information, including age, nationality, religion, marital status, duration of marriage, education level, work status, and social class. It also recorded participants’ district of residence, as geographical regions in Lebanon often reflect cultural and socioeconomic differences.^14^^,^^15^ Sexual and reproductive history was assessed, including history of sexual activity, frequency of intercourse, pregnancy status, number of children, and delivery mode. Also, factors such as FGM were included, considering their cultural and sexual health implications.^16^ FGM involves the partial or total removal of the external female genitalia for nonmedical reasons, typically performed during childhood or adolescence.^17^ Deeply rooted in cultural, religious, and social beliefs, especially in parts of Africa and the Middle East, it is prevalent in Egypt,^18^ where it is justified to enhance sexual purity, modesty, or prepare women for marriage. Despite being outlawed, FGM persists and is known to cause long-term health and sexual function complications for women.^16–20^

Following the sociodemographic survey, participants completed the culturally adapted FSFI-LB. The original FSFI questionnaire uses a Likert scale for scoring, with domain scores calculated by summing item responses and multiplying by domain-specific weighting factors. The total FSFI score ranges from 2 to 36, with higher scores indicating better sexual functioning. A cutoff score of 26.5 identifies potential sexual dysfunction.^21^ The FSFI demonstrates strong psychometric properties: high internal consistency (Cronbach’s alpha = 0.97 for the total score and alphas between 0.89 and 0.95 for domain scores), strong inter-rater reliability, construct validity, and excellent test–retest reliability. It has been widely validated across diverse populations, establishing it as a gold standard for identifying and quantifying FSD.

### Translation and cultural adaptation

The FSFI was adapted using a standardized back-translation method:

1. Forward translation: Two independent sworn translators fluent in English and Arabic translated the FSFI from English to Arabic.
2. Backward translation: Two other sworn translators fluent in English and Arabic translated the Arabic version back to English. Discrepancies were reconciled by comparing the back-translated version with the original.
3. Face validity: A pilot study involving 15 women with the inclusion criteria assessed clarity and cultural relevance. Feedback led to linguistic adjustments.
4. Expert panel review: A final version was approved by a panel comprising two gynecologists from HDF, a psychologist from Université Saint-Joseph de Beyrouth (USJ), and a professional translator. HDF is a university hospital affiliated with USJ, ensuring a collaborative environment that integrates clinical and academic expertise. This connection allowed the panel to provide a multidisciplinary review, combining medical, psychological, and linguistic perspectives to finalize the culturally adapted instrument.

### Statistical analysis

The statistical analysis was performed using R software (version 4.4.0).^22^ This version was utilized for data processing, performing descriptive and inferential statistics, and generating the necessary outputs for the analysis of the FSFI data.

### Factor analysis

- Given that the FSFI had not yet been validated in Lebanese Arabic, we used exploratory factor analysis (EFA) to investigate the underlying structure of the scale. EFA was preferred over confirmatory factor analysis (CFA) due to sample size limitations and to avoid conducting both analyses on the same dataset, which can lead to inflated model fit statistics. Factor retention decisions were guided by standard psychometric criteria, including Kaiser’s criterion (eigenvalues > 1), visual inspection of the scree plot, and clinical interpretability.^23–26^ The scree plot was generated by plotting the eigenvalues of each component in descending order to visually identify the “elbow,” where the curve begins to flatten, indicating the optimal number of factors to retain. Multiple factor solutions were explored to assess both statistical and conceptual alignment.^23^
- Bartlett’s test of sphericity was used to test whether the correlation matrix was suitable for factor analysis. It was conducted using the RedaS package to evaluate whether the variables in the dataset were sufficiently correlated for factor analysis.^27^
- The KMO measure and the measure of sampling adequacy (MSA) were used to evaluate sampling adequacy, ensuring the dataset was appropriate for factor analysis.^28^
- EFA was conducted using the MVar.pt package, with factor extraction and rotation procedures applied to identify latent dimensions of sexual function in the Lebanese context. Although “Oblimin” rotation was tested, the results were comparable to those obtained with “varimax”; hence, varimax rotation was retained for interpretability.^26^^,^^29^

### Reliability and validity

1. Internal consistency was measured using Cronbach’s alpha, a statistical measure used to evaluate the internal consistency of a scale, with a score of 0.70 or higher generally indicating good reliability.^30^
2. Pearson correlation was used to evaluate the relationships between the different factors of the questionnaire. This coefficient measures the strength and direction of the linear relationship between two sets of continuous variables, ranging from −1 (perfect negative correlation) to +1 (perfect positive correlation). Pearson’s correlation was chosen because the factors are continuous variables, and we aimed to assess linear associations between them.^31^
3. Test–retest reliability was used to assess the stability of the FSFI-LB over time. A subsample of participants were re-evaluated 4 weeks after their initial participation. The first 42 women who had completed the original questionnaire were contacted by telephone and invited to complete the FSFI-LB again. Those who did not answer were left with a voicemail or written message explaining the purpose of the follow-up. This subset was selected based on their early participation and availability for follow-up during the study timeline, making it feasible to apply the retest within a consistent interval. The 4-week interval was chosen to balance the need for temporal separation while minimizing the risk of significant changes in sexual function. Surveys were readministered online via SurveyMonkey to ensure privacy and convenience, using the same format and instructions. Scores from the first and second administration were compared using the Pearson correlation coefficient, providing a measure of temporal consistency.

### Cultural sensitivity and cutoff score

Like other studies,^11^^,^^32^^,^^33^ a cutoff score for the test was not applied due to the small sample size as it would not have provided reliable or valid results. The small sample size limited the ability to establish a meaningful threshold for classification; therefore, we opted to use an existing cutoff score from previously validated studies to classify sexual dysfunction. Among the available options, the cutoff score of 28.1 from the Egyptian version of the FSFI^7^ was selected, as it was established in a population with cultural and linguistic similarities to our study sample. This approach ensures consistency with validated research in the region and allows for comparability of results across studies.

## Results

### Content validity

The translation of the questionnaire into Classical Arabic presented several challenges related to the cultural and linguistic context of the Lebanese population. While Classical Arabic is often used in formal and academic contexts, its technical and medical terms—particularly those related to sexuality—proved difficult for many participants to understand. Words describing anatomical terms like “vagina” and physiological experiences like “orgasm” were either unfamiliar, elicited discomfort, or were perceived as disrespectful. Several participants expressed that certain terms were incomprehensible, while others responded with ironic laughter or verbalized their discomfort. In everyday conversations, especially when discussing topics related to sexuality, Lebanese people often prefer using English or French terms. To address these challenges and ensure content validity, the final version of the questionnaire included the English translation of certain terms alongside their Arabic counterparts (in parentheses). This approach significantly improved comprehension and reduced participants’ discomfort, as English terms for sexual health topics are more commonly understood and accepted in the Lebanese context. Furthermore, unlike other translated versions of the questionnaire that have used the term “husband” to translate “partner,” our study aimed to adopt a more inclusive approach. We used terminology that would resonate with a broader audience, including single and divorced women, for example, to better reflect the diverse marital statuses of our participants. This adaptation highlights the importance of considering cultural and linguistic nuances when developing tools for cross-cultural research, particularly on sensitive topics such as sexual health.

### Demographic characteristics

The demographic characteristics of the study sample (*N* = 119) are summarized in Table 1. The majority were aged between 18 and 45 (89.9%), with a predominance of Christians (51.3%), followed by Muslims (35.3%). Most participants were married (59.7%), and a significant portion had been married for less than 7 years (38.7%). Educationally, 40.3% held a bachelor’s degree, while 39.5% had a master’s or PhD. Employment status indicated that 76.5% were employed, and most participants identified as belonging to the middle social class (77.3%). Regarding sexual activity, 48.7% reported having sex 1-2 times per week. In terms of family dynamics, half of the participants had no children (50.4%), and the majority had not undergone FGM (99.2%). The delivery mode was predominantly cesarean (27.7%), with some participants reporting vaginal births (10.9%).

**Table 1:** Demographic characteristics of participants.

Characteristic	*n*	%
**Age**		
18-30	50	42.0
31-45	57	47.9
46-55	12	10.1
**Religion**		
Christian	61	51.3
Shia Muslim	22	18.5
Sunni Muslim	20	16.8
Druze	8	6.7
Atheist	8	6.7
**Marital status**		
Married	71	59.7
Single/Divorced/Separated/Widow	23	19.3
Engaged/In a relationship	25	21.0
**Marriage duration**		
A few months to 7 years	46	38.7
8 years and above	45	37.8
Not married	28	23.5
**Level of education**		
Elementary/High school	24	20.2
Bachelor’s degree	48	40.3
Master’s/PhD	47	39.5
**Current employment situation**		
Employed	91	76.5
Unemployed	28	23.5
**Social class**		
Low	15	12.6
Middle	92	77.3
High	12	10.1
**District**		
Mount Lebanon	62	52.1
Beirut	39	32.8
South Lebanese	6	5.0
North Lebanese	4	3.4
Akkar	4	3.4
Nabatyeh	3	2.5
Bekaa	1	0.8
**Frequency of sexual activity per week**		
Less than 1	34	28.6
1-2	58	48.7
3-4	24	20.2
More than 4	3	2.5
**Number of children**		
0	60	50.4
1	16	13.4
2	20	16.8
3 or more	17	14.3
Not specified	6	5.0
**Delivery mode**		
Did not give birth	61	51.3
**Vaginal birth**	13	10.9
Cesarean	33	27.7
**Both vaginal and cesarean**	7	5.9
Not specified	5	4.2
Underwent female genital mutilation		
Yes	1	0.8
No	118	99.2

### Statistical analysis

#### Test of sphericity

The test yielded a test statistic of 3121.322 with a *P*-value < 2.22 × 10^-16^, rejecting the null hypothesis that the correlation matrix equals the identity matrix. This result indicated significant correlations among variables, supporting the use of factor analysis.

#### Sampling adequacy

The KMO measure of sampling adequacy^28^ was excellent at 0.925, and the MSA values for all individual variables ranged from 0.843 to 0.967, confirming the data’s suitability for factor analysis. The lowest MSA was observed in question 16 (0.843), while the highest was in question 5 (0.962). This range is important to note as it highlights the varying degrees of relevance and consistency of each item in measuring the underlying construct, which may inform decisions about potential adjustments or exclusions in further analyses.

#### Factor analysis

**Figure 1:**
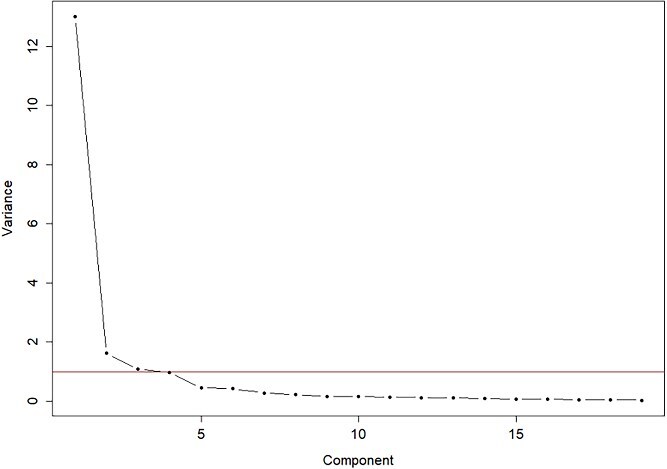
Scree plot of eigenvalues form the exploratory factor analysis.

Factor loadings from the EFA are presented in Tables 2 and 3. Table 3 provides a detailed summary of the factor structure. Based on Kaiser’s criterion (eigenvalues > 1), three factors were initially retained. To further support this decision, a scree plot was generated (Figure 1), which revealed a clear inflection point after the third component—indicating a potential three-factor solution. However, both five- and six-factor models were subsequently explored to assess clinical and theoretical alignment with the original FSFI structure. While the five-factor solution accounted for over 90% of the cumulative variance and showed high communalities across all items, the six-factor model was ultimately retained due to its superior clinical interpretability, particularly the cultural distinction between arousal and lubrication in the Lebanese context.

**Table 2:** Rotated factor loading matrix obtained via EFA (varimax rotation).

**Exploratory factor analysis using varimax rotation**						
	**Factor 1**	**Factor 2**	**Factor 3**	**Factor 4**	**Factor 5**	**Communality**
Q1	−0.290	0.093	−0.152	0.886	0.120	0.916
Q2	−0.256	0.180	−0.208	0.869	0.157	0.921
Q3	−0.609	0.296	−0.295	0.470	0.370	0.904
Q4	−0.566	0.318	−0.307	0.506	0.355	0.897
Q5	−0.607	0.224	−0.270	0.456	0.446	0.898
Q6	−0.600	0.310	−0.298	0.406	0.408	0.876
Q7	−0.769	0.267	−0.218	0.285	0.348	0.913
Q8	−0.780	0.363	−0.172	0.247	0.290	0.915
Q9	−0.781	0.309	−0.174	0.279	0.243	0.873
Q10	−0.785	0.361	−0.143	0.233	0.271	0.894
Q11	−0.475	0.238	−0.221	0.224	0.738	0.925
Q12	−0.549	0.291	−0.162	0.245	0.654	0.900
Q13	−0.455	0.295	−0.403	0.132	0.625	0.864
Q14	−0.664	0.344	−0.327	0.225	0.163	0.742
Q15	−0.269	0.154	−0.874	0.264	0.069	0.934
Q16	−0.172	0.104	−0.878	0.143	0.282	0.912
Q17	−0.271	0.886*	−0.111	0.181	0.195	0.941
Q18	−0.317	0.886	−0.095	0.073	0.145	0.922
Q19	−0.306	0.880	−0.167	0.174	0.182	0.959

**Table 3:** Retained factors: Eigenvalues, percentage of explained variance, and cumulative variance.

**Factor**	**Eigenvalue**	**Percentage of variance**	**Cumulative percentage**
Factor 1	13.006	68.452	68.452
Factor 2	1.611	8.480	76.932
Factor 3	1.079	5.679	82.611

#### Reliability analysis

The internal consistency of the questionnaire was assessed using Cronbach’s alpha, which yielded a value of 0.973, indicating excellent reliability. Despite the statistical support for a five-factor model, clinical considerations suggested a six-factor model consistent with Rosen et al. (2000). The first factor was divided into two domains: arousal and lubrication. Also, question 14, initially associated with factor 1, was reclassified under factor 3 (satisfaction) due to its moderate communalities (0.742) and weights across multiple factors. The *Desire* domain includes items 1 and 2, with scores ranging from 2 to 10. The *Arousal* domain consists of items 3, 4, 5, and 6, with a score range from 0 to 20. For the *Lubrication* domain, items 7, 8, 9, and 10 are considered, with scores ranging from 0 to 20. The *Orgasm* domain is represented by items 11, 12, and 13, with scores ranging from 0 to 15. The *Satisfaction* domain includes items 14, 15, and 16, with a score range from 2 to 15 (with a score of 0 or 1 possible for item 16). Finally, the *Pain* domain includes items 17, 18, and 19, with a score range from 0 to 15.

Significant positive correlations were observed between all factors, with values ranging from moderate to strong as shown in Table 4.

- The strongest correlations were observed between arousal and lubrication (*r* = 0.886), followed by arousal and orgasm (*r* = 0.867), indicating an intrinsic relationship between these physiological aspects of sexual function.
- Factors such as desire and pain showed the weakest correlation (*r* = 0.400), suggesting that sexual desire is less related to the experience of pain compared to other domains.
- Moderate correlations were observed between satisfaction and other factors (*r* ranging from 0.532 to 0.785), highlighting that sexual satisfaction is influenced by multiple domains but is not exclusively dependent on any single one.
- The correlations involving pain were consistently lower, with values ranging from 0.400 to 0.680, reflecting the potentially negative impact of pain on sexual function.

**Table 4:** Pearson correlation between factors.

Domain correlations						
	Desire	Arousal	Lubrification	Orgasm	Satisfaction	Pain
Desire	1	0.734	0.616	0.546	0.560	0.400
Arousal	0.734	1	0.886	0.867	0.785	0.644
Lubrification	0.616	0.886	1	0.846	0.702	0.680
Orgasm	0.546	0.867	0.846	1	0.729	0.620
Satisfaction	0.560	0.785	0.702	0.729	1	0.532
Pain	0.400	0.644	0.680	0.620	0.532	1

The Cronbach’s alpha values for each domain were calculated, with the Desire domain showing an alpha of 0.929, Arousal of 0.976, Lubrication of 0.971, Orgasm of 0.939, Satisfaction of 0.811, and Pain of 0.877. These values indicate excellent reliability for most domains, with Satisfaction exhibiting a slightly lower alpha but still considered acceptable. Each domain contained between 2 and 4 items, and the total sample size for the analysis was 114 participants.

These findings support the validity of the six-factor model, illustrating the interconnectedness of various domains of sexual function and their mutual influence. Also, based on the results presented, the sexual dysfunction most prevalent in the study sample appears to be related to sexual pain, especially since it showed less connection with other sexual function domains and had a notable impact on satisfaction and overall sexual health.

#### Test–retest reliability

The test–retest reliability was high for all domains (*r* > 0.99) and for the total scale (*r* = 0.9997), indicating excellent consistency across the measurements, as shown in Table 5.

**Table 5:** Test–retest reliability analysis.

**Test–retest reliability: *n* = 42**					
**Domains**	**Questions**	**Mean score test**	**Mean score retest**	**Pearson *r***	***P*-value**
Desire	1, 2	6.19	6.14	0.9928	<.001
Arousal	3-6	12.86	12.83	0.9976	<.001
Lubrification	7-10	13.60	13.60	0.9983	<.001
Orgasm	11-13	9.93	9.93	1.0000	<.001
Satisfaction	14-16	12.02	12.00	0.9977	<.001
Pain	17-19	11.36	11.33	0.9996	<.001
Total	1-19	65.95	65.83	0.9997	<.001

## Discussion

The FSFI-LB demonstrated excellent psychometric properties: Our study achieved strong internal consistency, with a Cronbach’s alpha of 0.973 consistent with the original FSFI’s alpha of 0.97.^6^ The KMO value of 0.925 indicated excellent sampling adequacy for factor analysis in line with Rosen et al.’s KMO of 0.88, supported by Bartlett’s test of sphericity, which was significant (*P* < .001). The tool also showed robust test–retest reliability (*r* > 0.99 for individual domains and *r* = 0.9997 for the total scale) aligning with Rosen et al.’s findings (2000), which reported a reliability range of *r* = 0.79-0.86. The original FSFI and the FSFI-LB demonstrate the tool’s stability in measuring sexual function, regardless of cultural or linguistic differences.

The translation of the questionnaire into Classical Arabic posed significant challenges due to cultural and linguistic sensitivities. Many participants found certain sexual health terms unclear, uncomfortable, or overly formal. To enhance clarity and participant comfort, we included English translations alongside specific Arabic terms and adopted inclusive terminology such as “partner” instead of “husband.” This approach was generally effective, especially among participants from urban and multilingual backgrounds who were familiar with English terminology commonly used in sexual health contexts. These adjustments highlight the importance of cultural sensitivity in sexual health research, influenced by factors such as limited sexual education and the impact of French and Western cultures in Lebanon.^34^ Our findings also align with previous studies that validated and culturally adapted the FSFI^7^^,^^10^^,^^11^^,^^32^^,^^33^^,^^35–39^ affirming that the FSFI-LB is a reliable and valid measure for assessing FSD in Lebanese women.

Although statistical indicators such as eigenvalues and the scree plot initially supported a five-factor model, we explored and ultimately retained a six-factor structure. This decision was guided by clinical input and cultural considerations—particularly the distinct perception of arousal and lubrication in the Lebanese context—and aligned with the original FSFI’s theoretical framework. In Lebanese society, where cultural and religious norms significantly shape sexual health discourse, women often describe arousal—understood as psychological or emotional readiness for sexual activity—as distinct from lubrication, a primarily physiological response.^40^ This approach challenges the growing trend of treating desire and arousal as nearly indistinct constructs, recognizing that such an overlap may not fully account for cultural nuances. Such a perspective aligns with previous findings indicating that cultural contexts influence how individuals interpret and report sexual function^41^^,^^42^ This decision also reflects a common methodological challenge in psychometric validation studies—balancing statistical fit with clinical and conceptual clarity.^24^^,^^25^ By separating arousal and lubrication, the FSFI-LB better captures these culturally specific distinctions, ensuring a more accurate and relevant assessment of sexual health for Lebanese women. This adjustment not only enhances the tool’s validity within this population but also underscores the importance of considering sociocultural factors in psychometric validation processes.

While the FSFI-LB demonstrates strong reliability and validity, there are important limitations that must be addressed in future research. First, the sample was composed entirely of Lebanese women, primarily recruited from a single hospital and through online outreach, which limits the generalizability of our findings. The current sample may overrepresent more educated, urban, and sexually open individuals, potentially excluding more conservative women or those with lower digital access. To enhance generalizability and applicability across Lebanon’s diverse population, future studies should employ stratified sampling strategies that encompass rural areas, multiple religious communities, various socioeconomic strata, and underrepresented governorates.

This study also presents limitations related to sample composition. To ensure psychometric homogeneity in this initial validation phase, we excluded pregnant women and individuals over the age of 55, as both pregnancy and menopause introduce distinct hormonal and physiological changes that can significantly affect sexual function.^43^^,^^44^ However, we acknowledge that this exclusion limits the tool’s applicability in clinical settings, particularly in assessing sexual dysfunctions such as dyspareunia or pain during intercourse, which are highly prevalent among postmenopausal women. Future validation studies should specifically include pregnant and postmenopausal populations to evaluate how the FSFI-LB performs in these groups and to determine whether modifications or adjusted scoring thresholds are necessary.

Also, the use of convenience and snowball sampling may have introduced selection bias. Participants who are more open to discussing sexual health, more educated, or more sexually active may be overrepresented in the current sample, while women from more conservative backgrounds or with limited sexual experience may be underrepresented. This limitation may affect the representativeness of the results and the applicability of the FSFI-LB across the broader population. To address this in future studies, we recommend implementing community-based recruitment strategies, partnering with public health centers and women’s organizations in both urban and rural areas, and using stratified sampling techniques to ensure better demographic diversity across education levels, religiosity, and sexual experience.

Language-related challenges also represent a key limitation of this study. The strategy of using English alongside Arabic may not have fully addressed the needs of all the participants—particularly those with limited English proficiency or those from more conservative backgrounds. The inclusion of English may have inadvertently introduced a barrier to comprehension, and the taboo nature of certain sexual terms may have led to underreporting or biased responses due to discomfort, embarrassment, or perceived social judgment. These issues highlight the need for future qualitative research, including cognitive interviews and focus groups, to better understand participants’ interpretations, emotional reactions, and potential misunderstandings when completing the FSFI-LB. Future versions of the tool could also explore regional dialectal adaptations, term glossaries, or simplified wording, ensuring accessibility and cultural sensitivity across the diverse linguistic and social spectrum of the Lebanese population.

Moreover, given the cultural, linguistic, and dialectal diversity within the Arab world, further comparative validation studies are necessary across different Arabic-speaking countries. These studies should examine the FSFI-LB’s performance in other regional contexts (eg, North Africa, the Gulf, and the Levant). Such cross-national validation efforts will help determine the tool’s broader cultural relevance, support the development of region-specific cutoff scores, and foster standardized assessment practices in Arabic sexual health research.

Although the pain domain demonstrated weaker correlations with other FSFI-LB domains—particularly with desire and satisfaction—it was retained due to its essential clinical role in diagnosing conditions such as dyspareunia and vaginismus, which are often underrecognized in both research and practice, particularly in conservative sociocultural settings. The distinct nature of pain as a sexual dysfunction warrants its separation, even if its statistical alignment with other domains is less robust. Its inclusion allows for the identification of specific dysfunction patterns that would otherwise be obscured in a merged or overly simplified model. However, we recognize that the lower interdomain correlations may signal challenges in how pain-related items are interpreted by participants. Future research should include qualitative exploration, such as cognitive interviews, to better understand how women in Lebanon perceive and report sexual pain, and whether linguistic, cultural, or emotional factors affect the domain’s clarity and measurement precision.

It is important to address that this study did not include CFA, which is considered the gold standard for assessing model fit. Due to the limited sample size (*N* = 119), conducting both EFA and CFA on the same dataset would have introduced bias and compromised the validity of the factor structure. Future studies should validate the FSFI-LB using CFA in an independent, larger sample to confirm the stability and fit of the proposed factor model.^24^^,^^25^

The FSFI-LB was not compared to formal clinical diagnoses of FSD, which limits our ability to assess its diagnostic accuracy. While we applied a previously validated cutoff score (28.1) from the Egyptian version to identify potential cases of dysfunction, this approach is provisional and context-dependent. Cultural, linguistic, and demographic differences between populations may affect the appropriateness of this threshold in the Lebanese context. Future research should include structured clinical interviews, ideally based on the Diagnostic and statistical manual of mental disorders, fifth edition (DSM-5) criteria, to evaluate the sensitivity, specificity, and predictive validity of the FSFI-LB. Such studies are essential for establishing clinically relevant cutoff scores that can reliably distinguish between women with and without diagnosable sexual dysfunction in Lebanon and other Arabic-speaking populations.

Finally, this study did not evaluate convergent or discriminant validity between FSFI-LB domains and other psychological constructs, such as anxiety or depression. Future validation studies should assess these forms of validity using a priori hypotheses and ideally a separate, larger sample, to further refine the scale’s psychometric properties and construct validity in relation to psychological and sexual health outcomes.

When comparing our study to the Egyptian FSFI developed by Anis et al. (2011), several cultural and methodological differences become apparent. Both studies aimed to culturally adapt and validate the FSFI in Arabic-speaking populations, but the cultural contexts of Lebanon and Egypt differ significantly. Anis et al.’s study focused on Egyptian women, a population where the Muslim majority, comprising approximately 90% of the population,^45^ significantly shapes cultural and religious norms, emphasizing modesty and the moral dimensions of sexuality. In contrast, Lebanon’s population is more religiously diverse, with Muslims making up about 69.3% and Christians around 30.7%.^46^ This demographic balance leads to a broader range of cultural perspectives, influenced by both Eastern and Western values. Our study is the first to consider a higher proportion of Christian women than Muslim women in the context of sexuality in Arabic-speaking countries and the Middle East, providing a more diverse representation of religious backgrounds. In addition, while Anis et al.’s study concentrated on married women, our study included a more diverse sample, encompassing not only married women but also single women, as well as those who are widowed, separated, divorced, or engaged. As a result, the ways in which women in Lebanon perceive and discuss sexual issues are shaped by this mix of cultural influences and a broader range of life experiences, creating a different context for sexual health and attitudes compared to Egypt. The women in our study were from a broader range of cultural, religious, and social backgrounds, providing a more inclusive perspective.

Moreover, the prevalence of FGM in Egypt, with 62.2% of Anis et al.’s participants reporting the procedure, plays a significant role in shaping women’s sexual experiences. The high prevalence in their study influences how Egyptian women respond to questions on sexual function.^18^^,^^20^ This may complicate an accurate assessment of sexual health, as FGM is linked to a range of physical and psychological consequences, such as painful intercourse, reduced sexual satisfaction, and emotional distress.^19^ In contrast, Lebanon, while sharing some cultural and religious traditions with Egypt, has a significantly lower prevalence of FGM, with only one woman in our study reporting having undergone the procedure. Lebanon’s more diverse and pluralistic religious and cultural environment has led to a lower incidence of FGM. However, Lebanon still faces challenges in sexual health, largely due to conservative religious attitudes and the complex interplay of cultural norms, but FGM is not a widespread issue.

Furthermore, the linguistic adaptations in our study highlight important distinctions from those employed by Anis et al. While their approach exclusively utilized Arabic terms, our study integrated both Arabic and English terminology to accommodate the unique linguistic landscape of Lebanon, which is deeply influenced by its bilingual and, to some extent, trilingual population. This decision was rooted in the observation that Lebanese participants often encounter discomfort or confusion when engaging with Classical Arabic terms for sexual health, due to limited sexual education and the influence of Western languages such as English and French in everyday discourse. By including English terms alongside their Arabic counterparts, we aimed to ensure clarity and relatability, thereby enhancing participants’ understanding and willingness to engage with the questionnaire.

This difference in linguistic strategy may have significantly influenced how participants interpreted specific questions and reported their sexual function. In contrast to Anis et al., our dual-language approach may have reduced misinterpretation and encouraged more accurate reporting by aligning the questionnaire’s language with the participants’ lived experiences. To further elaborate, the integration of more inclusive terminology—such as using “partner” instead of “husband”—allowed us to capture a broader range of marital statuses, ensuring that single, divorced, or widowed women were not excluded from the discourse on sexual health. These adaptations emphasize the critical role of culturally and linguistically sensitive methodologies in cross-cultural research, particularly in regions like Lebanon, where language and cultural diversity significantly shape communication.^2^^,^^3^^,^^47^^,^^48^

The FSFI-LB addresses a significant gap in sexual health assessment tools for Lebanon, where sexual health is often underdiscussed due to cultural and religious sensitivities.^49^ This validated tool, the FSFI-LB, provides healthcare professionals with a more accurate means of assessing FSD, offering valuable insights into sexual health concerns and enhancing the quality of care. The tool’s development is timely, given Lebanon’s urgent need for improved sexual health education and resources. The FSFI-LB paves the way for future research and interventions, increasing the awareness and reducing the stigma surrounding sexual health.

The tool’s applicability extends beyond Lebanon to other Arabic-speaking populations in the Middle East, where cultural, linguistic, and religious factors similarly influence sexual health perceptions. It provides a model for adapting sexual health assessment tools in these regions, ensuring they are culturally and linguistically appropriate. By validating the FSFI-LB, we empower Lebanese women, especially those from socially excluded groups, by providing them with a reliable tool to address their sexual health concerns. This contributes to their overall well-being and promotes a more inclusive healthcare approach.

This study highlights the need for enhanced sexual health education and resources for women in Lebanon, particularly those from socially excluded groups who may have limited access to such education.^50^ By validating culturally sensitive tools like the FSFI-LB, healthcare professionals can better address the sexual health needs of these women. This research not only advances sexual health knowledge but also paves the way for a more inclusive and culturally aware healthcare approach. Further research is needed to refine the tool, establish valid cutoff scores, and explore the impact of cultural, religious, and regional factors on sexual health outcomes in Lebanon.

## Conclusion

The FSFI-LB demonstrated strong psychometric properties, confirming its validity and reliability for assessing female sexual function in Lebanon. By maintaining the original FSFI’s six-factor structure and incorporating culturally sensitive linguistic adaptations, it ensures diagnostic accuracy and conceptual relevance. This validated tool provides clinicians and researchers with a standardized instrument tailored to Lebanon’s sociocultural landscape, while also offering a model for future adaptations across the Arab world. Beyond its clinical utility, the FSFI-LB represents an important step toward reducing stigma and fostering open, informed conversations about female sexual health in conservative settings. Future studies should establish clinical cutoff scores and pursue comparative validation in other Arabic-speaking populations, strengthening the regional understanding of sexual dysfunction and enhancing inclusive patient care.

## Supplementary Material

FSFI-LB_qfaf055
