# Supplementary material for: Translation, cultural adaptation, and psychometric validation of the Female Sexual Function Index to Lebanese Arabic (FSFI-LB)
Source: Sex Med. 2025 Aug 5;13(4):qfaf055. doi: 10.1093/sexmed/qfaf055 (PMC12358250; doi:10.1093/sexmed/qfaf055)
Supplement: FSFI-LB_qfaf055 [file fsfi-lb_qfaf055.pdf]

Appendix 1: FSFI-LB Final Version

مؤشّر الوظائف الجنسية عند المرأة

التعليمات:
يرجى الإجابة على الأسئلة التالية استنادًا إلى تجاربك خلال *الأربعة أسابيع الماضية* .هذه الأسئلة تتعلق بوظيفتك الجنسية وتجربتك، والتي قد تشمل أفكارك ومشاعرك وسلوكياتك الجنسية. أجب على كل سؤال بأكبر قدر ممكن من الدقة. لا توجد إجابات صحيحة أو خاطئة. يرجى اختيار الرقم الذي يعكس تجربتك بشكل أفضل. إذا لم يكن السؤال ينطبق عليك، يرجى اختيار "0" لذلك البند.
لكل سؤال، يرجى الإشارة إلى الإجابة التي تعكس بشكل أكثر دقة تجربتك خلال الأربعة أسابيع الماضية. إذا لم يكن لديك أي نشاط جنسي أو لم تكن نشطة جنسيًا خلال الأسابيع الأربعة الماضية، يرجى الانتقال إلى نهاية الاستبيان واختيار الخيار الذي يعكس حالتك بشكل أفضل.
كل سؤال يحتوي على مقياس للإجابات التي تتوافق مع مستوى تجربتك خلال الفترة الزمنية المحددة. ستوفر إجاباتك معلومات حول التكرار، والشدة، والرضا المتعلق بالجوانب المختلفة لوظيفتك الجنسية.

Instructions:
"Please answer the following questions based on your experiences during the *last four weeks*. These questions are related to your sexual function and experience, which may include your sexual thoughts, feelings, or behaviors. Answer each question as accurately as possible. There are no right or wrong answers. Please circle the number that best reflects your experience. If a question does not apply to you, please choose "0" for that item.
For each question, please indicate the most accurate answer based on your experiences during the last 4 weeks. If you have not had any sexual activity or have not been sexually active during the past four weeks, please skip to the end of the questionnaire and select the option that best represents your situation.
Each question has a scale of answers that corresponds to your level of experience during the specified time period. Your responses will provide information about the frequency, intensity, and satisfaction related to various aspects of your sexual function

السؤال	خيارات الردّ/الإجابة
1. خلال الأسابيع الأربعة الماضية، كم مرةٍ شعرت بالرغبة الجنسي (sexual desire) أو الاهتمام الجنسيّ (sexual interest) ؟	5 = على الدوام تقريبًا أو دائمًا<br>4 = معظم الأوقات (أكثر من نصف الوقت)<br>3 = في بعض الأحيان (حوالي نصف الوقت)<br>2 = بضع مرات (أقلّ من نصف الوقت)<br>1 = أبدًا تقريبًا أو أبدًا
2. خلال الأسابيع الأربعة الماضية، كيف يمكنك تقييم مستوى (درجة) رغبتك الجنسية (sexual desire) أو اهتمامك الجنسيّ (sexual interest)؟	5 = عالٍ جدًّا<br>4 = عالٍ<br>3 = متوسط/معتدل<br>2 = منخفض<br>1 = منخفض جدًا أو معدوم<br>0 = لا نشاط جنسي على الإطلاق
3. خلال الأسابيع الأربعة الماضية، كم مرّةٍ شعرت بالإثارة الجنسية(sexual arousal) أثناء النشاط أو الجماع الجنسيّ (sexual activity or intercourse) ؟	5 = على الدوام تقريبًا أو دائمًا<br>4 = معظم الأوقات (أكثر من نصف الوقت)<br>3 = في بعض الأحيان (حوالي نصف الوقت)<br>2 = بضع مرات (أقلّ من نصف الوقت)<br>1 = أبدًا تقريبًا أو أبدًا<br>0 = لا نشاط جنسي على الإطلاق
4. خلال الأسابيع الأربعة الماضية، كيف يمكنك تقييم مستوى (درجة) شعورك بالإثارة	5 = عالٍ جدًّا

الجنسية (sexual arousal) أثناء النشاط أو الجماع الجنسيّ (sexual activity or intercourse)؟

4 = عالٍ
3 = متوسط/معتدل
2 = منخفض
1 = منخفض جدًا أو معدوم

5. خلال الأسابيع الأربعة الماضية، كم كنت واثقة بأنك ستشعرين بالإثارة الجنسية (sexual arousal) أثناء النشاط أو الجماع الجنسيّ (sexual activity or intercourse)؟

0 = لا نشاط جنسي على الإطلاق
5 = ثقة عالية جدًّا
4 = ثقة عالية
3 = ثقة معتدلة
2 = ثقة منخفضة
1 = ثقة منخفضة أو معدومة

6. خلال الأسابيع الأربعة الماضية، كم مرةٍ كنت راضية عن الإثارة الجنسية (sexual arousal) التي شعرت بها أثناء ممارسة النشاط الجنسي أو الجماع (sexual activity or intercourse)؟

0 = لا نشاط جنسي على الإطلاق
5 = على الدوام تقريبًا أو دائمًا
4 = معظم الأوقات (أكثر من نصف الوقت)
3 = في بعض الأحيان (حوالي نصف الوقت)
2 = بضع مرات (أقلّ من نصف الوقت)
1 = أبدًا تقريبًا أو أبدًا

7. خلال الأسابيع الأربعة الماضية، كم مرةٍ شعرت بأنك أصبحت رطبة/مبللة (lubricated/wet) خلال النشاط أو الجماع الجنسيّ (sexual activity or intercourse)؟

0 = لا نشاط جنسي على الإطلاق
5 = على الدوام تقريبًا أو دائمًا
4 = معظم الأوقات (أكثر من نصف الوقت)
3 = في بعض الأحيان (حوالي نصف الوقت)
2 = بضع مرات (أقلّ من نصف الوقت)
1 = أبدًا تقريبًا أو أبدًا

8. خلال الأسابيع الأربعة الماضية، كم مرةٍ كان من الصعب عليك أن تصبحي رطبة/مبللة(lubricated/wet) خلال النشاط أو الجماع الجنسيّ (sexual activity or intercourse)؟

0 = لا نشاط جنسي على الإطلاق
1 = صعب للغاية أو مستحيل
2 = صعب جدًا
3 = صعب
4 = قليل الصعوبة
5 = ليس صعبًا

9. خلال الأسابيع الأربعة الماضية، كم مرة استطعت أن تحافظي على الرطوبة أو البلل (lubrication or wetness) إلى حين إكمال النشاط أو الجماع الجنسيّ (sexual activity or intercourse)؟

0 = لا نشاط جنسي على الإطلاق
5 = على الدوام تقريبًا أو دائمًا
4 = معظم الأوقات (أكثر من نصف الوقت)
3 = في بعض الأحيان (حوالي نصف الوقت)
2 = بضع مرات (أقلّ من نصف الوقت)
1 = أبدًا تقريبًا أو أبدًا

10. خلال الأسابيع الأربعة الماضية، كم مرةٍ كان من الصعب عليك أن تحافظي على الرطوبة أو البلل (lubrication or wetness) إلى حين إكمال النشاط أو الجماع الجنسيّ (sexual activity or intercourse)؟

0 = لا نشاط جنسي على الإطلاق
1 = صعب للغاية أو مستحيل
2 = صعب جدًا
3 = صعب
4 = قليل الصعوبة
5 = ليس صعبًا

11.خلال الأسابيع الأربعة الماضية، وعندما كان لديك التحفيز الجنسي (sexual stimulation) أو الجماع الجنسي (sexual intercourse)، كم مرةٍ وصلت إلى النشوة الجنسية (orgasm) ؟	0 = لا نشاط جنسي على الإطلاق<br>5 = على الدوام تقريبًا أو دائمًا<br>4 = معظم الأوقات (أكثر من نصف الوقت)<br>3 = في بعض الأحيان (حوالي نصف الوقت)<br>2 = بضع مرات (أقلّ من نصف الوقت)<br>1 = أبدًا تقريبًا أو أبدًا
12.خلال الأسابيع الأربعة الماضية، وعندما كان لديك التحفيز الجنسي (sexual stimulation) أو الجماع الجنسي (sexual intercourse)، هل كان من الصعب عليك أن تصلي إلى النشوة الجنسية (orgasm) ؟	0 = لا نشاط جنسي على الإطلاق<br>1 = صعب للغاية أو مستحيل<br>2 = صعب جدًا<br>3 = صعب<br>4 = قليل الصعوبة<br>5 = ليس صعبًا
13.خلال الأسابيع الأربعة الماضية، إلى أيّ درجةٍ كنت راضية من تمكنك من الوصول إلى النشوة الجنسية (orgasm) خلال النشاط أو الجماع الجنسي (sexual activity or intercourse)؟	0 = لا نشاط جنسي على الإطلاق<br>1 = راضية جدًّا<br>2 = راضية باعتدال<br>3 = راضية وغير راضية على السواء<br>4 = غير راضية باعتدال<br>5 = غير راضية على الإطلاق
14.خلال الأسابيع الأربعة الماضية، إلى أيّ درجةٍ كنت راضية من كمية التقارب العاطفي والحميم الذي اختبرته خلال النشاط الجنسي (sexual activity) بينك وبين شريكك؟	0 = لا نشاط جنسي على الإطلاق<br>1 = راضية جدًّا<br>2 = راضية باعتدال<br>3 = راضية وغير راضية على السواء<br>4 = غير راضية باعتدال<br>5 = غير راضية على الإطلاق
15.خلال الأسابيع الأربعة الماضية، إلى أيّ درجةٍ كنت راضية من علاقتك الحميمة والجماع بينك وبين شريكك؟	5 = راضية جدًّا<br>4 = راضية باعتدال<br>3 = راضية وغير راضية على السواء<br>2 = غير راضية باعتدال<br>1 = غير راضية على الإطلاق
16.خلال الأسابيع الأربعة الماضية، إلى أيّ درجةٍ كنت راضية من حياتك الجنسية بالإجماع؟	5 = راضية جدًّا<br>4 = راضية باعتدال<br>3 = راضية وغير راضية على السواء<br>2 = غير راضية باعتدال<br>1 = غير راضية على الإطلاق
17.خلال الأسابيع الأربعة الماضية، كم مرةٍ شعرت بالانزعاج أو بالألم خلال الإدخال المهبلي (vaginal penetration)؟	0 = لم نحاول الجماع<br>5 = على الدوام تقريبًا أو دائمًا<br>4 = معظم الأوقات (أكثر من نصف الوقت)<br>3 = في بعض الأحيان (حوالي نصف الوقت)<br>2 = بضع مرات (أقلّ من نصف الوقت)<br>1 = أبدًا تقريبًا أو أبدًا

0 = لم نحاول الجماع

18.خلال الأسابيع الأربعة الماضية، كم مرةٍ شعرت بالانزعاج أو بالألم ما بعد الإدخال المهبلي (vaginal penetration) ؟

5 = على الدوام تقريبًا أو دائمًا
4 = معظم الأوقات (أكثر من نصف الوقت)
3 = في بعض الأحيان (حوالي نصف الوقت)
2 = بضع مرات (أقلّ من نصف الوقت)
1 = أبدًا تقريبًا أو أبدًا
0 = لم نحاول الجماع

19.خلال الأسابيع الأربعة الماضية، كيف يمكنك تقييم مستوى (درجة) شعورك بالانزعاج أو بالألم خلال أو بعد الإدخال المهبلي (vaginal penetration)؟

5 = عالٍ جدًّا
4 = عالٍ
3 = متوسط/معتدل
2 = منخفض
1 = منخفض جدًا أو معدوم
